# Exploring the relationship between methylation of the cortisol receptor genes and brain and cognitive outcomes in individuals with elevated amyloid-β

**DOI:** 10.21203/rs.3.rs-7540786/v1

**Published:** 2025-09-10

**Authors:** Tenielle Porter, Ayeisha Milligan Armstrong, Lidija Milicic, Michael Vacher, Shane Fernandez, Eleanor O’Brien, Vincent Doré, Pierrick Bourgeat, Rosita Shishegar, Ying Xia, Christopher Rowe, Victor Villemagne, Paul Maruff, Colin Masters, Giuseppe Verdile, Simon Laws, David Groth

**Affiliations:** Edith Cowan University; National Center for Posttraumatic Stress Disorder; Edith Cowan University; Edith Cowan University; Austin Health; CSIRO; Austin Health; MHRI, University of Melbourne, Melbourne Australia/CogState, Melbourne; 4The Florey Institute of Neuroscience and Mental Health, The University of Melbourne; McCusker Alzheimer’s Research Foundation; Edith Cowan University

## Abstract

Chronic stress has been implicated as a risk factor for Alzheimer’s disease (AD), although the molecular pathways linking stress with AD are poorly understood. In this study, relationships between differential methylation of the cortisol receptor genes, the glucocorticoid and mineralocorticoid receptors (*NR3C1* and *NR3C2*, respectively) and brain-health phenotypes (cognition, brain amyloid-b (Aβ) burden and regional brain volumes) were examined in two independent cohorts of cognitively unimpaired individuals with accumulating brain Aβ. In addition, interactions between methylation and depression/anxiety symptoms were assessed. Nominal associations between methylation and brain-health phenotypes, including cognition, brain Aβ burden, and regional brain volumes, were observed across both cohorts. Further analyses of interactions between *CpG x depression/anxiety symptoms* identified that the relationships between methylation and brain-health outcomes differed significantly depending on the presence of depression/anxiety symptoms. Among the observed associations, lower methylation at *cg24052866 (NR3C1)* was associated with faster cognitive decline, whilst lower methylation at *cg07275757 (NR3C2), cg10993059 (NR3C2)*, and *cg25708981 (NR3C1)* was associated with faster brain atrophy, specifically in those individuals with depressive symptoms. These results suggest that across the four CpG sites lower methylation is associated with worse outcomes in those with depression symptoms. This work has identified relationships between methylation of cortisol receptor genes and AD phenotypes, many of these moderated by depression symptoms. These findings, highlight the need for further investigation into methylation as biomarkers for stress-related risk in AD.

## Introduction

Chronic psychological stress can have a lasting negative impact on bodily systems [1–3]. One molecular mechanism proposed to explain these lasting effects is stress-induced alterations to epigenetic profiles [4]. Substantial evidence from animal and human studies shows that stress exposure can lead to epigenetic changes, particularly DNA methylation in peripheral and brain tissues, resulting in altered gene expression (as reviewed in [5]). Epigenome-wide DNA methylation changes in peripheral blood monocyte cells (PBMCs) are consistently reported in chronically-stressed populations (reviewed in [4, 5]). In addition, *in vitro* exposure of hippocampal progenitor cells to glucocorticoids alters DNA methylation at ~ 30,000 cytosine-phosphate-guanine (CpG) sites [6]. Whilst methylation at these CpG sites within transcription start sites commonly represses transcription or silences genes, the role of gene-body methylation remains to be fully elucidated, with evidence suggesting possible involvement in transcription elongation or splicing [7]. The main glucocorticoid hormone in humans is cortisol, produced in response to stress, which binds to both the glucocorticoid (GR) and mineralocorticoid receptors (MR) to exert effects throughout the body [8]. The GR and MR are nuclear transcription factor proteins, regulating target gene expression [9–11]. It is possible that epigenetic changes to the genes that encode these receptors may lead to downstream changes in the modulation of gene expression. It is proposed that changes to DNA methylation via exposure to adversity or chronic stress in early-life may result in altered stress reactivity in tissues later in life by altering the thresholds of transcriptional changes induced by stress [6]. In conjunction with epigenome-wide effects, studies have also used targeted approaches investigating methylation profiles of stress-related genes including the GR gene (*NR3C1*) [12].

Chronic stress in early to mid-life is associated with an increased risk of Alzheimer’s disease (AD) in later life [13–15]. Furthermore, anxiety and depression have been identified as AD risk factors [16–18]. It has been suggested that the aetiology underpinning the association between depression symtoms and AD is unlikely due simply to an increase in brain amyloid-β (Aβ) [19]. High levels of self-reported anxiety or plasma cortisol levels accelerate cognitive decline specifically in individuals who are classified cognitively unimpaired (CU) who also have abnormal brain Aβ [20, 21]. Chronic stress is also a risk factor for depression and anxiety [22, 23] highlighting the complex interplay between stress, neuropsychiatric disorders, and neurodegenerative processes. Furthermore, stress, particularly in early-life, induces long-lasting DNA methylation changes [4], which may represent a plausible biological mechanism linking AD risk from stress, depression/anxiety and age. Studies have reported associations between differences in DNA methylation and AD diagnosis or related phenotypes (as reviewed in [24]). Importantly, these methylation associations have been observed in tissues outside of the brain, including PBMCs. Therefore, changes in methylation patterns in PBMCs as a result of exposure to glucocorticoids could serve as a surrogate for glucocorticoid-induced changes in the brain [25]. However, tissue-specific changes in methylation patterns occur and therefore limits the interpretation of peripheral methylation associations, as they neither infer causality nor reveal functional effects for brain-health phenotypes [26]. Despite this, accessing brain tissue of living participants is not feasible, and therefore PBMC methylation studies may assist in identifying biomarkers or serve to develop risk profiles for brain-related phenotypes.

It is known that chronic stress can alter the methylation patterns of genes, particularly the cortisol receptor genes (*NR3C1/NR3C2*). Therefore, the overall aim of this study was to assess the relationships between differential methylation at CpG sites within the genes encoding the two cortisol receptors, the GR and MR, (*NR3C1/NR3C2*) and differences in brain-health phenotypes (Aβ, cognition and brain volumes). This study also aimed to assess *CpG methylation x depression/anxiety symptom* interactions and their association with brain-health phenotypes. Given cortisol and anxiety was previously seen to alter cognitive decline specifically in those who were CU with abnormal brain Aβ [20, 21], the current study undertook analyses using data from two independent, well-characterised observational cohort studies, focusing on CU individuals with accumulating brain Aβ.

## Methods

### Study Participants

Participant data from the Australian Imaging Biomarker and Lifestyle (AIBL) https://aibl.org.au/ study of ageing, and the Alzheimer’s Disease Neuroimaging Initiative (ADNI) www.adni-info.org cohort studies [27–30] was used. Both are large, longitudinal studies aimed at identifying biomarkers and risk factors for AD. AIBL, based in Australia, was designed to complement ADNI by following similar protocols, allowing for cross-cohort comparisons. For this analysis participants were limited to those that were classified as CU at baseline as determined by thorough neuropsychological assessments spanning multiple cognitive domains in each respective cohort [27, 28, 31], but showed evidence of brain Aβ accumulation. Brain Aβ accumulation was defined using the baseline Centiloid (CL) score (described below) of > 20 or a positive CL slope across a minimum of three assessments. Participants were limited to those with methylation data available. Sample sizes varied across analyses due to outcome-specific data availability [Supplementary Table 1]. Importantly, whilst the presence of neuropsychiatric conditions at baseline were exclusion criteria at recruitment for both cohorts participants were not excluded from either study if they record clinically significant Hospital Anxiety and Depression Scale (HADS) and/or Geriatric Depression Scale (GDS) scores at later time-points. The AIBL study is approved by ethics committees at St Vincent’s Health, Hollywood Private Hospital, Edith Cowan University, and CSIRO. ADNI has ethics approval from all Institutional Review Boards where data was collected over the course of the study. Ethics approval was also provided by Curtin University for the current analyses.

### Stress Trait Measures

AIBL participants completed the HADS questionnaires and the 15-item GDS (GDS-15) [32–34] at multiple timepoints. Only GDS-15 scores [34] were available for analysis of ADNI participants. When examining depression and anxiety symptoms in interaction with CpG site methylation, the cohorts were dichotomized to explore the relationship between clinically significant scores and brain-health phenotypes. Specifically a clinically significant score was considered when an individual met established clinical thresholds of ≥8 for HADS depression or anxiety [35] or ≥5 for GDS depression [36, 37] at any visit.

### Brain Imaging

Positron emission tomography (PET) with tracers targeting Aβ were used to quantify brain Aβ levels in both AIBL and ADNI. Aβ PET scans were analysed using CapAIBL software to generate standardised uptake value ratios (SUVR) for the different tracers used [38]. These tracer specific SUVR levels were then transformed and expressed in Centiloids [39, 40].

Participants from AIBL and ADNI underwent magnetic resonance imaging (MRI) using the T1-weighted magnetization-prepared rapid gradient echo (MPRAGE) protocol to measure brain volumes. Volumetric measures of brain tissues and structures, including grey matter, white matter, ventricles, and hippocampi, were calculated using the CurAIBL pipeline as detailed in prior studies [41–43]. Brain volume measurements were adjusted for total intracranial volume and the specific MRI scanner used.

### Cognitive Testing

Participants from AIBL and ADNI underwent extensive neuropsychological evaluation using their respective neuropsychological test batteries [27–30, 44, 45]. For the AIBL cohort, composite scores for five cognitive domains were calculated including episodic recall, recognition memory, executive function, language, attention and processing speed [44], as well as the Preclinical Alzheimer’s Cognitive Composite (PACC) [45]. For the ADNI cohort, only the PACC was used to define global cognitive performance [45]. The AIBL-PACC and ADNI-PACC had been constructed previously to evaluate the same cognitive domains (episodic memory, timed executive function, and global cognition) using the different, but highly related, neuropsychological tests [45].

### Methylation Data

DNA from AIBL and ADNI participants was isolated from whole blood using QIAamp DNA blood spin column kits (Qiagen, Valencia, CA, USA) [27, 28, 30]. DNA methylation was determined as previously described [31, 46]. Briefly, bisulfite conversion was performed using a EZ DNA Methylation Kit (Zymo Research, Orange, CA, USA) followed by genome-wide DNA methylation profiling on the Infinium HumanMethylation EPIC (850k) BeadChip array (Illumina, Inc., San Diego, CA, USA). Quality control and normalisation of DNA methylation data was undertaken using the “*meffil*” package [47] in R. Quality control measures included removing samples exhibiting technical artifacts, low-quality probes or samples based on P-value detection, insufficient bead counts, improper methylated/unmethylated signal ratios, and dye biases. Control probe checks were conducted, as well as the identification of sample swaps using SNP discordance between methylation and genotype arrays, and verification of sex, all performed using the “*meffil*” package [47]. CpG sites were filtered out of the analysis if there was a single nucleotide polymorphism located either at the CpG site, or within the probe sequences. The samples underwent quantile normalisation using the dasen method in the “*wateRmelon*” package [48]. The *Houseman* method [49], implemented in “*meffil*”, was used to predict PBMC cell composition (*B cells, CD4T, CD8T, eosinophil, monocyte, neutrophil, natural killer cells*) for each sample.

CpG sites within the *NR3C1/NR3C2* genes (GR/MR) ±10 kbp regions were analysed. Methylation at each site was represented using beta (β) values (range 0–1). Samples with β methylation values >10 SD from the mean were removed as outliers (AIBL = 24; ADNI = 7). Sites with mean β methylation values >0.95 or <0.05, or methylation ranges <0.05, were excluded. This left 92 CpG sites for AIBL and 103 for ADNI.

### Genotyping

In AIBL, the participants’ *Apolipoprotein E* genotype (*APOE*) was determined using TaqMan^®^ genotyping assays (Life Technologies, USA) on the Quant-StudioTM 12k Flex Real-Time-PCR system (Applied BiosystemsTM, USA), following manufacturer’s instructions [27, 28, 30]. In ADNI, *APOE* genotypes were assessed via PCR amplification and *HhaI* restriction enzyme digestion [50, 51]. *APOE-ε4* status was analysed as a binary variable (presence/absence of an *ε4* allele).

### Statistical Analysis

All statistical analyses were undertaken with R Statistical Software version 4.3.2 [52], using RStudio (2024.12.1.563) [53]. Demographic descriptive statistics (mean and standard deviations) were compiled for AIBL and ADNI participants overall, as well as for the subsets included in the different outcome variable analyses.

For cross-sectional analyses, the outcome measure was taken from the same time-point at which methylation data was collected, or within 18 months. These contemporaneous time-points then served as the baseline for assessing the relatopnships between baseline methylation levels and longitudinal changes in the brain-health phenotypes. When cognition or brain volumes were assessed in longitudinal analyses, linear mixed models with random slopes and intercepts were utilised within the “*lme4*” package [54] to estimate each participants’ intercept (representative of baseline) and slope values (over a minimum of three assessments) for each outcome variable of interest. Estimated slope values were used as the outcome variable for longitudinal analyses, with the intercept value used as a covariate. Slopes of change for brain Aβ were calculated using a least-squares linear regression fitted to the data points available (minimum of three assessments) for each participant [55].

Linear regression analyses using the *lm* function in R were used to assess the relationships between 1.) the level of methylation at CpG sites and brain-health phenotypes (brain Aβ-burden, cognitive measures, brain volume measures) both cross-sectionally and longitudinally, and 2.) the interaction between the *level of methlation at CpG sites x depression/anxiety symptoms* and brain-health phenotypes, both cross-sectionally and longitudinally. For interaction analyses, HADS and GDS measures were dichotomised separately based on those who had ever scored above eight or five respectively, and those that had not. For all analyses age, sex, smoking status (known to significantly alter methylation [56]) and PBMC cellular compositions, *APOE-ε4* status (binary) and years of formal education (only when assessing cognition) were included as covariates. For longitudinal analyses, participants’ intercept values for the given traits were also included as covariates.

Nominal signficiance was set at an uncorrected p threshold of p < 0.05. Correction for multiple testing was performed using the False Discovery Rate (FDR) method [57], where p values for the terms of interest (main effect or interaction) were adjusted within each outcome variable per cohort (q value). Whilst associations that remain significant following FDR correction represent the most robust findings, an association observed at the nominal significance threshold across two independent cohorts or analyses also provides credible evidence of an effect, particularly when the direction and magnitude of the association are consistent. Therefore whilst all results in the current study are reported, those associations that either reached FDR significance, or were nominally significant across multiple outcome measures across the two independent cohorts, with consistent directions of effect are discussed in detail.

## Results

### Demographics

Group demographic statistics (AIBL *n = 305* and ADNI *n* = 102) are summarised in Table 1. ADNI participants were on average older (~ 2 years) than AIBL participants. A similar finding for age remained in the sub-population analyses by outcome variable, where it was also observed that ADNI participants had more years of formal education on average than AIBL participants [Supplementary Table 1].

### Selection of CpG methylation sites

Following quality control measures, 92 CpG sites for AIBL and 103 CpG sites for ADNI (90 in common) were taken through to analysis. The location of these CpG sites, along with their mean and range of methylation values in the two cohorts, are shown in Supplementary Table 2.

#### CpG sites within GR/MR genes (NR3C1/NR3C2) are nominally associated with differences in cognition assessed longitudinally.

Whilst nominal associations between methylation at CpG sites and measures of cognition assessed cross-sectionally were found in both AIBL and ADNI cohorts, none of these associations remained significant following FDR correction [Table 2]. In addition, none of these findings were in common between AIBL and ADNI cohorts.

**Figure 1:**
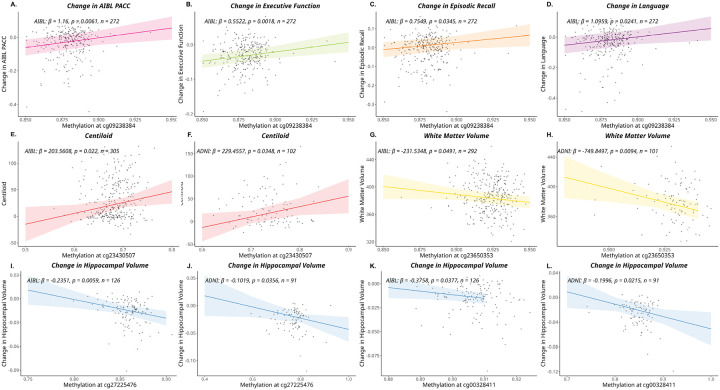
Direct associations between CpG site methylation and brain-health phenotypes. Linear regression results depicting associations between DNA methylation and cognitive or neuroimaging outcomes. Methylation at cg09238384 was associated with changes in cognitive performance across **(A)**PACC, **(B)** executive function, **(C)** episodic recall, and **(D)**language domains within the AIBL cohort. Associations between methylation at cg23430507 and Centiloid were observed in **(E)** AIBL and **(F)** ADNI. Relationships between methylation at cg23650252 and white matter volume were identified in **(G)** AIBL and **(H)** ADNI. Differences in the rate of hippocampal atrophy were associated with methylation at cg27225476 in **(I)**AIBL and **(J)** ADNI, and with cg00328411 in **(K)** AIBL and **(L)**ADNI. The coefficient if represented as β, the p value represents the significance of the CpG methylation level wth the outcome of interest. Covariates: age, sex, *APOE-ε4*status, smoking status, cellular composition (B cell, CD4T, CD8T, eosinophil, monocyte, neutrophil, natural killer cells), education (for cognition only) and participants intercept value (for longitudinal outcomes only). Abbreviations: AIBL, Australian Imaging Biomarkers and Lifestyle Study; ADNI, Alzheimer’s Disease Neuroimaging Initiative; APOE, apolipoprotein E; PACC, Pre-clinical Alzheimer’s Cognitive Composite.

For longitudinal analyses of cognition, nominal associations were observed; however once again none of these remained significant following FDR correction [Table 2]. Of the nominal associations that were observed across multiple outcomes, lower methylation at one CpG site cg09238384, located within the MR gene-body (*NR3C2*), was associated with faster rates of cognitive decline in four of the six domains tested in AIBL: AIBL-PACC, executive function, episodic recall and language cognitive domains [Figure 1]. Across the four cognitive composites, the model predicted a 10% lower methylation at cg09238384 corresponded with an average of 0.116, 0.055, 0.075 and 0.110 SD/year faster decline in cognitive function for AIBL-PACC, executive function, episodic recall and language cognitive domains, respectively. No other consistent associations across multiple cognitive outcomes were observed.

#### CpG sites within the GR/MR genes (NR3C1/NR3C2) are nominally associated with differences in brain imaging measures.

Nominal associations between methylation at CpG sites and brain Aβ-burden (CL) and regional brain volumes analysed both cross-sectionally and longitudinally were observed in both AIBL and ADNI cohorts, however none of these associations remained significant following FDR correction [Table 2; Supplementary Table 3 for full results]. Amongst the statistical signals passing the nominal threshold, it was found that higher methylation at cg23430507, located within the *NR3C1* gene-body, was associated with higher cross-sectional brain Aβ levels in both AIBL and ADNI cohorts [Figure 1]. Here, a 10% increase in methylation at cg23430507 was associated on average with a 20.35 and 22.94 CL units higher Aβ burden in AIBL and ADNI, respectively. A difference in 20 CL units can be considered a large effect, given 20–25 CL is typically the threshold between being classified as Aβ low or high [58, 59]. No other associations overlapped across both cohorts for the Aβ analyses.

Nominal associations between methylation status at CpG sites and differences in cross-sectional measures of all four regional brain volumes measured were observed in both AIBL and ADNI cohorts, but again, no associations were significant following FDR correction [Table 2]. When comparing the results between the two cohorts, one CpG site cg23650353, located downstream of *NR3C1*, was associated with white matter volume in both AIBL and ADNI cohorts. Specifically, 10% higher methylation at cg23650353 on average corresponded with a 23.1cm^3^ and 74.9cm^3^ smaller white matter volumes in the cross-sectional setting in AIBL and ADNI participants, respectively [Figure 1]. While this framing facilitates biological interpretation, it is important to note that the actual range of methylation at this site is narrow (~ 10%), being 86–95% in AIBL and 85–95% in ADNI.

In longitudinal analyses, nominal associations were observed between methylation at CpG sites and all regional brain volumes examined. When assessing the overlap of these between the cohorts, it was found that two CpG sites were associated with differences in hippocampal volumes in both AIBL and ADNI cohorts in consistent directions of effect [Figure 1]. Higher methylation at the two sites, cg00328411 and cg27225476, both located within *NR3C2* gene-body, was associated with an increased rate of hippocampal atrophy in both the AIBL and ADNI cohorts [Figure 1]. A 10% higher methylation level at cg00328411 was associated with an additional average loss of 0.037 cm^3^/year (1.28% of average hippocampal volume) and 0.020 cm^3^/year (0.67%) in hippocampal volume in the AIBL and ADNI cohorts, respectively. Similarly, a 10% higher methylation at cg27225476 was associated with an additional average hippocampal volume loss of 0.023 cm^3^/year (0.79%) in AIBL and 0.010 cm^3^/year (0.34%) in ADNI. These effects can be considered moderate given typical hippocampal atrophy rates for preclinical AD are between 0.06–0.17cm^3^/year [60, 61] and the reported differences in hippocampal atrophy rates between cognitively unimpaired individuals with and without Aβ is 0.099 cm^3^/year [62].

#### The interactions of CpG sites x stress-related traits are associated with differences in cognition.

**Figure 2:**
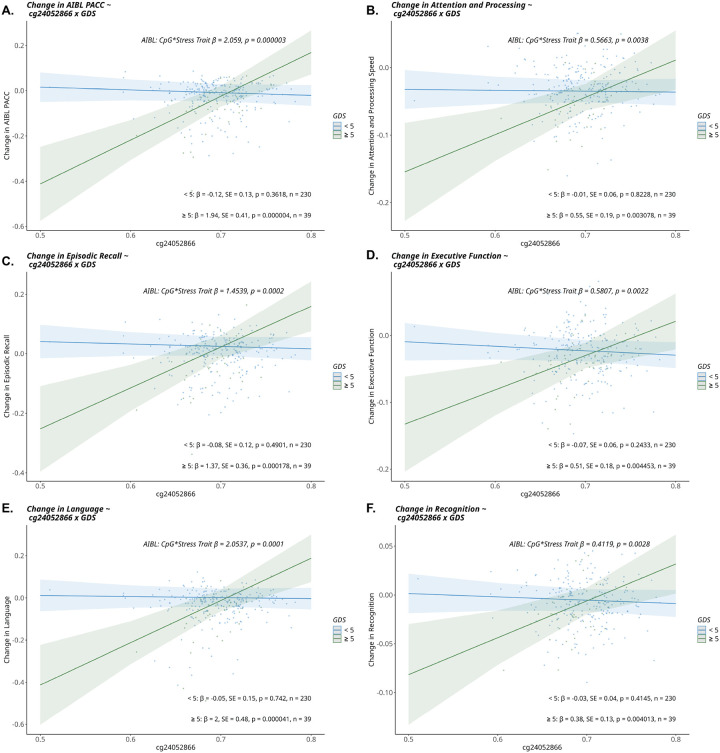
Linear regression results of cg24052866 x GDS interaction term with cognition in AIBL Linear regression results modelling the interaction term of cg24052866 x GDS and its association with cognition assessed longitudinally in AIBL. Longitudinal slope values (y-axis) were calculated based on a minimum of three cognitiveassessments. The cohort was dichotomised into those that had ever recorded a score ≥ 5 on the GDS, representing presence of depressive symptoms, and those that had not. In those that had scored ≥ 5 in GDS, methylation at cg24052866 was associated with rates of cognitive decline in **(A)**AIBL-PACC, **(B)** attention and processing, **(C)** episodic recall, **(D)**executive function, **(E)** language and **(F)** recognition cognitive domains. The interaction term is represented along with its associated p value, as is the estimated marginal means and associated p values of the GDS group specific effects. Covariates: baseline age, intercept trait value, sex, *APOE-ε4* status, education, smoking status, cellular composition (B cell, CD4T, CD8T, eosinophil, monocyte, neutrophil, natural killer cells). Abbreviations: AIBL, Australian Imaging Biomarker and Lifestyle Study; GDS, Geriatric Depression Scale; PACC, Pre-clinical Alzheimer’s disease Cognitive Composite.

Linear regression analyses revealed nominally significant *methylation at CpG sites x HADS scores* interaction effects, indicating that the relationships between methylation and cognition differed depending on HADS scores within the AIBL cohort for both cross-sectional and longitudinal analyses [Supplementary Table 4]. This was also observed with interactions between the *methylation at CpG sites x GDS* and measures of cognition, in both the cross-sectional and longitudinal settings in both AIBL and ADNI cohorts [Supplementary Table 4], however FDR significant associations were identified in the AIBL cohort only [Table 3]. When reviewing the CpG sites that were associated with multiple cognitive domains within AIBL, it was found that *cg24052866* (located within the gene-body of *NR3C1*) x *GDS* was associated with all domains tested. Specifically, for those that had ever recorded a GDS score of ≥ 5, less methylation at cg24052866 was associated with increased rates of cognitive decline [Figure 2; Supplementary Table 4]. This indicates that in those who had recorded a clinically significant GDS score, a 10% lessening in methylation at cg24052866, was associated with a 0.194, 0.055, 0.137, 0.051, 0.200 and 0.038 SD/year increase in cognitive decline for AIBL-PACC, attention and processing speed, episodic recall, executive function, language and recognition domains, respectively. These associations reached FDR significance levels for three cognitive domains (AIBL-PACC, episodic recall and language) [Table 3]. There was no overlap of *CpG site* x *stress-trait* associations with equivalent outcome variables between AIBL and ADNI cohorts.

#### The interactions of CpG sites x stress-related traits are associated with differences in brain imaging measures.

**Figure 3:**
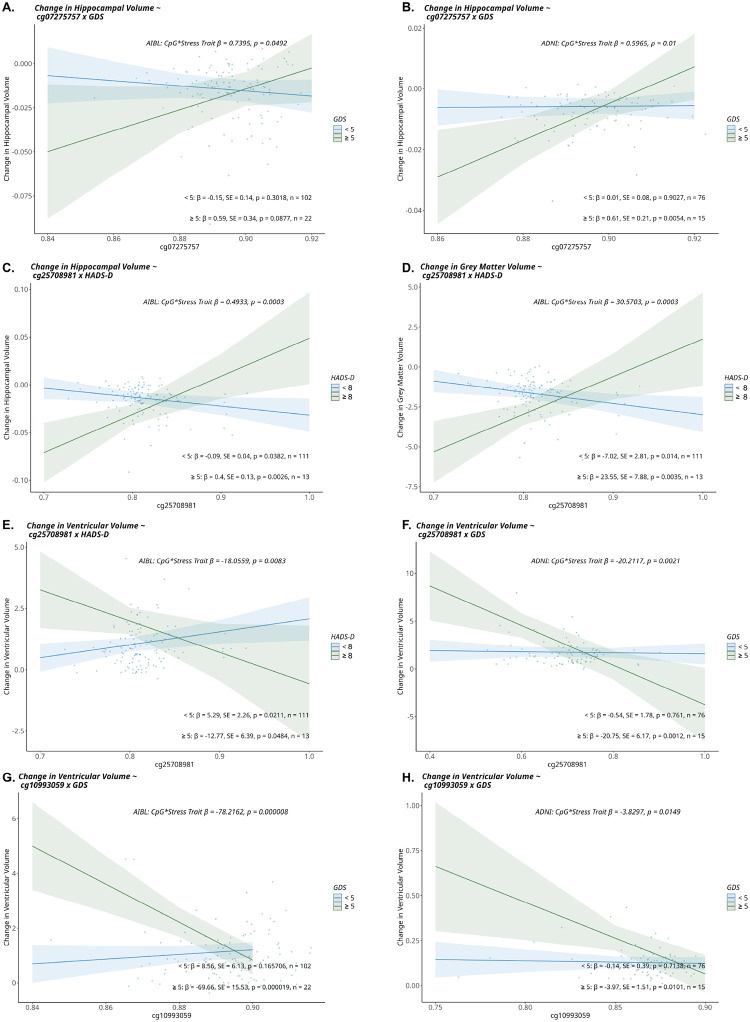
Linear regression results of CpG x GDS interaction term with brain imaging measures. Linear regression results modelling interactions between DNA methylation and depression scores in relation to neuroimaging outcomes. The interaction between cg07275757 × GDS was associated with hippocampal atrophy in **(A)** AIBL and **(B)** ADNI. The interaction between cg25708981 × HADS-D was associated with longitudinal changes in **(C)** hippocampal volume, **(D)** grey matter volume, and **(E)** ventricular volume in AIBL. In ADNI, an association was observed between cg25708981 × GDS and ventricular volume change **(F)**. Finally, the interaction between cg10993059 × GDS and longitudinal changes in ventricular volume was associated in **(G)** AIBL and **(H)**ADNI. The cohorts were dichotomised into those that had ever recorded a score ≥ 5 on the GDS or ≥8 for HADS-D, representing presence of depressive symptoms, and those that had not. The interaction term is represented along with its associated p value, as is the estimated marginal means and associated p values of the GDS group specific effects. Covariates: baseline age, intercept trait value, sex, *APOE-ε4* status, smoking status, cellular composition (B cell, CD4T, CD8T, eosinophil, monocyte, neutrophil, natural killer cells). Abbreviations: AIBL, Australian Imaging Biomarker and Lifestyle Study; ADNI, Alzheimer’s Disease Neuroimaging Initiative; GDS, Geriatric Depression Scale; HADS-D, Hospital Anxiety and Depression Scale - Depression.

Interaction analyses revealed that the relationships between methylation at CpG sites with Aβ-burden and regional brain volumes significantly differed depending on the presence of the stress-related traits. For Aβ-burden nominal associations were observed for both AIBL and ADNI cohorts [Supplementary Table 4]. Whilst for regional brain volumes many FDR significant relationships were observed (as denoted by the q value) [Table 3], along with some cohort-dependent findings of the same CpG site nominally associated with multiple volume measures [Supplementary Table 4]. Of particular interest were the findings where the same CpG site was associated with brain volume measures in both the AIBL and ADNI cohorts. In longitudinal analyses, the interaction of *NR3C2* gene-body *CpG site cg07275757 x GDS* was nominally associated with differences in hippocampal atrophy in both AIBL and ADNI cohorts [Figure 3; Supplementary Table 4]. Among participants who had ever scored ≥ 5 on the GDS, lower methylation at cg07275757 was associated with faster hippocampal atrophy. Every 10% decrease in methylation corresponded to an increase of 0.059 cm^3^/year (2.04%) in AIBL and 0.061 cm^3^/year (2.05%) in ADNI. Based on previously described rates these represents moderate effect sizes [60–62]. In the AIBL cohort, the CpG site cg25708981 within *NR3C1* interacted with *HADS-D* and was associated with hippocampal and grey matter atrophy as well as ventricular expansion, with all associations reaching FDR significance [Figure 3; Table 3]. In individuals scoring ≥ 8 on the HADS-D, a 10% lower methylation at cg25708981 corresponded to faster hippocampal atrophy (0.04 cm^3^/year, 1.38%), greater grey matter atrophy (2.35 cm^3^/year, 0.51%), and larger ventricular expansion (1.27 cm^3^/year, 3.4%). Additionally in ADNI, cg25708981 interacted with GDS to predict ventricular expansion at FDR significance; participants scoring ≥ 5 on the GDS showed 2.07 cm^3^/year (5.18%) faster ventricular enlargement for every 10% lower methylation [Figure 3; Table 3]. Given previously described hippocampal atrophy rates discussed above, in addition to reported grey matter atophy rates being 3.39–6.99 cm^3^/year [60, 63] and ventricular increases being 3.20–3.52 cm^3^/year [61, 64] in those with preclinical AD or MCI, the associations reported here represent a moderate effect size. Finally, a third site cg10993059 in NR3C2 interacted with GDS and was associated with ventricular volume change in both AIBL and ADNI. Among participants scoring ≥ 5 on the GDS, lower methylation at cg10993059 was associated with faster ventricular volume expansion corresponding to an average increase of 6.9cm^3^/year (18.7%) in AIBL and 0.3cm^3^/year (1.0%) in ADNI, reaching FDR significance in AIBL and nominal significance in ADNI [Figure 3; Supplementary Table 4].

## Discussion

This study identified consistent relationships between differential methylation at individual CpG sites within the GR/MR genes (*NR3C1/NR3C2*) and brain-health phenotypes in two independent cohorts with accumulating brain Aβ. Although the direct associations between methylation and the brain-health phenotypes did not reach FDR significance, multiple associations were validated across the two cohorts at the nominal level, which provides evidence of a reproducible relationship. Moreover, *CpG methylation* interacted with *HADS/GDS*, indicating that depression and anxiety symptoms may moderate these relationships with brain-health.

Despite none of the direct associations between CpG site methylation and brain-health outcomes reaching FDR level significance, several of the nominal associations either validated across the two cohorts or were associated with multiple outcomes. These will form the focus of the following discussion. When examining cognition, cg09238384 within *NR3C2* was of particular interest due to its association with multiple outcome measures. Lower levels of methylation at cg09238384 were associated with increased rates of cognitive decline in the AIBL cohort. This CpG site has not been previously reported in relation to brain phenotypes in any prior studies. However in addition to its associations with cognitive decline reported here, lower methylation at this CpG site was also nominally associated with smaller cross-sectional white matter and hippocampal volumes in AIBL and ADNI, respectively. Collectively the results suggest an overall negative effect of lower methylation at cg09238384 on markers of brain health.

When assessing brain Aβ, higher methylation at the cg23430507 site within *NR3C1* was associated with higher Aβ-burden in both the AIBL and ADNI cohorts. Previously, in those with major depressive disorder, increased methylation of this CpG site in PBMCs was associated with more severe decoupling and disruption of structural connectivity and functional connectivity (SC-FC) in the brain [65]. This is of interest because SC-FC coupling is known to be reduced by chronic stress and additionally is associated with worse cognition, and neuropsychiatric and neurodegenerative conditions, including AD [66–68]. As the association of this site in the current study was found cross-sectionally, no conclusions regarding the direction of effect can be made. However, these findings raise the possibility that increased methylation at this site could be associated with worsening neurodegenerative processes via reduced SC-FC as a result of higher Ab burden, though this remains to be tested in future studies.

For regional brain volume measures, three CpG sites (cg23650353, cg00328411 and cg27225476) within *NR3C2* showed consistent associations across both cohorts. Firstly, greater methylation at cg23650353 was associated with smaller white matter volume in both AIBL and ADNI cohorts. In addition, for rates of hippocampal volume atrophy, greater methylation at cg00328411 was associated with increased rates of atrophy in both cohorts. To the best of our knowledge, neither of these sites have been identified in the literature to date. At another site (cg27225476), higher methylation was associated with increased hippocampal atrophy across both AIBL and ADNI cohorts. This CpG site has been previously associated with differences in levels of circulating proteins related to brain health [69].

Having found direct associations between CpG methylation sites and brain-health phenotypes, interaction analyses were undertaken to understand the role of depression/anxiety symptoms in moderating the association of methylation at *CpG sites* with brain-health phenotypes. For cognition, one site *NR3C1 cg24052866* interacted with depression symptoms (GDS) to be associated with all cognitive domains tested in AIBL. Specifically, in those that had ever recorded clinically significant depressive symptoms, lower methylation was associated with increased rates of cognitive decline. This site is located in an open sea region within the *NR3C1* and has not previously been implicated in brain phenotypes.

For brain volume measures three CpG sites were of interest when comparing the overlapping results between cohorts. Two of the three (*cg07275757 and cg10993059 within NR3C2)* have not been previously reported to be associated with brain phenoptypes. The *cg07275757 x GDS* interaction was associated with hippocampal atrophy, whereas the *cg10993059 x GDS* interaction was associated with differences in longitudinal measures of ventricular volume in both AIBL and ADNI cohorts. The interaction between a third site, (cg25708981 within *NR3C1)* and measures of depression symptoms (HADS-D in AIBL and GDS in ADNI) was associated with multiple regional longitudinal brain volumes across both cohorts and survived FDR correction, highlighting its significance. Within AIBL higher methylation at this site was also nominally associated directly with smaller cross-sectional hippocampal volumes (10% increase in methylation corresponding to 0.137 cm^3^ smaller volumes). This site is of particular interest as higher methylation at this CpG in PBMCs has also been previously associated with smaller hippocampal volumes in a paediatric population (mean age 5.13 years old) [70]. Furthermore, higher methylation at this site has been observed in individuals with social anxiety who experienced childhood adversity, as compared to those who had experienced childhood advserity without developing social anxiety [71]. Methylation at the cg25708981 site has also been reported to mediate the relationship between social anxiety disorder and brain activity in the context of early-life adversity [71]. In addition, methylation of this site has been shown to be reduced in buccal cells following exposure to dexamethasone, a NR3C1 agonist [72], suggesting sensitivity to stress. Our findings suggest that in the presence of depressive symptoms (interaction analyses), higher methylation at the cg25708981 site is protective in the context of brain atrophy, which is seemingly in contrast to our other finding (direct association analysis) in AIBL that higher methylation is associated with smaller hippocampal volumes. However, it has been suggested that increased methylation at cg25708981 following adversity may be an adaptation to repeated exposure to stress [71], and our findings reflect a role for the interaction between this site and depression symptoms. Although early-life adversity data are unavailable for AIBL and ADNI participants, its potential influence on later-life brain volume warrants further investigation.

The current study provides evidence that differences in methylation at CpG sites within the cortisol receptor genes are associated with differences in brain-health phenotypes. While the direct effects were limited to nominal associations only, robust interaction results passed the FDR threshold and these support a role for depressive symptoms in particular in moderating these effects. An observation made from these findings is that in most cases the associations between methylation and the brain-health phenotypes were stronger in those individuals with higher depressive symptoms, suggesting that these people may be more sensitive to the effects of methylation. In addition, of the CpG sites discussed, lower levels of methylation in PBMCs in those with depressive symptoms was associated with worse measures of brain health. From the current study no conclusions regarding causality or mechanisms can be made. However, a prior study of PBMC *NR3C1* methylation found that depression moderated methylation effects on acute coronary syndrome outcomes [73], supporting the role of depression moderating the impact of methylation on disease. Depression and the hypothalamic-pituitary-adrenal (HPA) axis, the major stress response system regulating cortisol, are intricately linked biologically [23]. Consequently, altered methylation of cortisol receptors (*NR3C1/NR3C2*) and depressive symptoms may act synergistically on biological pathways, contributing to the observed associations. Since this study shows depressive symptoms can modulate methylation effects, it is plausible that other factors may similarly influence these relationships.

Discussed above are those associations that showed consistent directions of effects across multiple outcomes or cohorts. However, certain CpG sites exhibited discordant directions of association between AIBL and ADNI, which may be attributable to underlying variability or noise within the datasets or modulating effects of confounding factors. A recognised limitation of this study is the lack of lifetime stress exposure data which can alter DNA methylation (as reviewed in [4–6]), and potentially modulate its effects. Future studies on other cohorts would be beneficial in understanding how these associations may differ in the context of differing exposures to lifetime stress, pathologies and symptomatic disease. Other study limitations must also be acknowledged. Firstly, methylation data was derived from bisulfite sequencing, which, whilst being common practice, has inherent limitations and has been shown to introduce technical bias [74]. However, as outlined in the methods above, appropriate quality control measures were employed to minimise these effects. In addition, DNA methylation profiles were taken from whole blood PBMCs, not brain tissue and whilst there have been some sites that correlate between the two reported, many do not [26]. Therefore, no causative nor functional conclusions can be made. However, this study provides evidence that peripheral markers in the blood are associated with brain health measures, which is an important finding.

In conclusion, this study identified novel associations between differential methylation at CpG sites within cortisol receptor genes and brain-health phenotypes in two independent cohorts with accumulating brain Aβ. Although many associations were only nominally significant, several replicated across analyses or cohorts, strengthening the findings. For most CpG sites, this is the first reported association with a brain-related phenotype. Future studies incorporating environmental factors such as lifetime stress exposure are needed to interrogate these relationships. Given that chronic stress can alter DNA methylation (as reviewed in [4–6]), investigating methylation in other stress-related genes may help identify peripheral biomarkers of vulnerability to stress-driven neurodegeneration.

## Supplementary Material

Supplementary Files

This is a list of supplementary files associated with this preprint. Click to download.

- 2025MRGRMethylationSupplementary05092025.xlsx

## Figures and Tables

**Table 1:** Demographics of AIBL and ADNI cohorts classified as cognitively unimpaired Aβ accumulators with DNA methylation available.

Variable	ADNI	AIBL	p
**n**	102	305	
**Age mean(sd)**	74.8 (6.1)	73.2 (6.3)	**0.03**
**Sex**			1
**Male**	43(42.2%)	128(42%)	
**Female**	59(57.8%)	177(58%)	
**APOE-ε4**			0.57
**ε4−**	70(68.6%)	198(64.9%)	
**ε4+**	32(31.4%)	107(35.1%)	
**Centiloid mean(sd)**	27.3 (36.3)	29.3 (36)	0.63

Demographics of AIBL and ADNI participants taken at baseline timepoint. Abbreviations: *APOE, Apolipoprotein E* genotype represented as binary of presence or absence of the *ε4* allele; IVC, intracranial volume corrected; HADS, Hospital Anxiety and Depression Scale; GDS, Geriatric Depression Scale; sd, standard deviation.

**Table 2:** CpG sites nominally associated with brain-health phenotypes.

Study	Brain Health Phenotype			CpG	n	β	95% CI	p	q	Mean Methylation	SD	Region	Location Build37
**AIBL**	**Cognition**	**PACC**	**CS**	cg10207656	303	6.984	[0.413–13.556]	**0.038**	0.710	0.909	0.01	Open Sea	chr4:149019526
				cg04867484	303	−4.732	[−8.819–0.645]	**0.024**	0.710	0.062	0.03	Open Sea	chr4:149297469
				cg10002915	303	−0.991	[−1.898–0.084]	**0.033**	0.710	0.542	0.08	S Shelf	chr4:149369175
				cg03746860	303	−7.185	[−14.029–0.341]	**0.041**	0.710	0.895	0.01	Open Sea	chr5:142759375
				cg14438279	303	2.783	[0.24–5.326]	**0.033**	0.710	0.807	0.06	Open Sea	chr5:142806343
			**Long**	cg09238384	272	1.160	[0.338–1.982]	**0.006**	0.560	0.880	0.01	Open Sea	chr4:149358199
				cg16219186	272	0.608	[0.078–1.138]	**0.025**	0.887	0.885	0.02	N Shore	chr5:142780531
				cg08423118	272	−0.147	[−0.284–0.009]	**0.037**	0.887	0.775	0.07	Open Sea	chr5:142808610
		**Attention and Processing**	**CS**	cg21701890	303	3.874	[0.02–7.728]	**0.050**	0.458	0.616	0.03	Open Sea	chr4:149094632
				cg26081259	303	−12.778	[−23.692–1.863]	**0.022**	0.414	0.926	0.01	Open Sea	chr5:142659682
				cg13514002	303	8.269	[0.47–16.068]	**0.039**	0.445	0.910	0.01	Open Sea	chr5:142677292
				cg19645279	303	3.307	[0.614–5.999]	**0.017**	0.414	0.506	0.03	Open Sea	chr5:142702733
				cg22233604	303	−4.365	[−8.584–0.145]	**0.044**	0.445	0.802	0.03	Open Sea	chr5:142729377
				cg03857453	303	−3.026	[−5.547–0.505]	**0.019**	0.414	0.718	0.05	Open Sea	chr5:142729913
				cg16535116	303	−4.756	[−9.106–0.406]	**0.033**	0.445	0.824	0.02	Open Sea	chr5:142769612
				cg19432243	303	1.881	[0.307–3.454]	**0.020**	0.414	0.752	0.06	Open Sea	chr5:142770757
				cg00294552	303	3.906	[0.2–7.613]	**0.040**	0.445	0.842	0.02	N Shore	chr5:142780486
				cg12466613	303	15.302	[2.627–27.977]	**0.019**	0.414	0.917	0.01	Open Sea	chr5:142815469
			**Long**	cg17253842	272	−0.296	[−0.57–0.022]	**0.035**	0.846	0.871	0.02	Open Sea	chr4:149347991
				cg12741214	272	0.576	[0.058–1.094]	**0.030**	0.846	0.922	0.01	Open Sea	chr5:142695619
				cg16535116	272	0.214	[0.003–0.425]	**0.048**	0.846	0.824	0.02	Open Sea	chr5:142769612
		**Episodic Recall**	**CS**	cg10207656	303	9.779	[1.907–17.651]	**0.016**	0.476	0.909	0.01	Open Sea	chr4:149019526
				cg04867484	303	−6.314	[−11.213–1.415]	**0.012**	0.476	0.062	0.03	Open Sea	chr4:149297469
				cg16586394	303	9.423	[0.803–18.042]	**0.033**	0.759	0.909	0.01	Open Sea	chr5:142757011
				cg07742588	303	−2.919	[−5.253–0.584]	**0.015**	0.476	0.789	0.06	N Shore	chr5:142780439
			**Long**	cg09238384	272	0.755	[0.059–1.451]	**0.035**	0.793	0.880	0.01	Open Sea	chr4:149358199
				cg16224829	272	−0.327	[−0.577–0.078]	**0.011**	0.793	0.792	0.04	Open Sea	chr5:142792698
				cg23430507	272	−0.416	[−0.78–0.051]	**0.026**	0.793	0.679	0.04	Open Sea	chr5:142798375
				cg23776787	272	−0.983	[−1.831–0.135]	**0.024**	0.793	0.052	0.02	Open Sea	chr5:142814315
		**Executive Function**	**CS**	cg02471166	303	3.549	[0.143–6.955]	**0.042**	0.966	0.629	0.02	Open Sea	chr4:149244985
				cg03857453	303	−2.691	[−5.099–0.282]	**0.029**	0.966	0.718	0.05	Open Sea	chr5:142729913
				cg07742588	303	−2.963	[−5.082–0.844]	**0.007**	0.601	0.789	0.06	N Shore	chr5:142780439
			**Long**	cg13000004	272	−0.375	[−0.737–0.013]	**0.043**	0.795	0.870	0.01	Open Sea	chr4:149074093
				cg27234800	272	−0.073	[−0.139–0.008]	**0.030**	0.680	0.891	0.05	Open Sea	chr4:149116611
				cg06240648	272	0.526	[0.057–0.995]	**0.029**	0.680	0.909	0.01	Open Sea	chr4:149289762
				cg09238384	272	0.552	[0.208–0.896]	**0.002**	0.170	0.880	0.01	Open Sea	chr4:149358199
				cg08423118	272	−0.070	[−0.128–0.012]	**0.018**	0.680	0.775	0.07	Open Sea	chr5:142808610
		**Language**	**CS**	cg27460943	303	5.940	[0.678–11.201]	**0.028**	0.850	0.083	0.03	N Shore	chr4:149362809
				cg16535116	303	−5.412	[−10.176–0.647]	**0.027**	0.850	0.824	0.02	Open Sea	chr5:142769612
				cg23776787	303	−12.289	[−21.925–2.652]	**0.013**	0.850	0.052	0.02	Open Sea	chr5:142814315
			**Long**	cg19650300	272	1.188	[0.311–2.065]	**0.008**	0.370	0.872	0.01	Open Sea	chr4:149357960
				cg09238384	272	1.096	[0.149–2.043]	**0.024**	0.370	0.880	0.01	Open Sea	chr4:149358199
				cg10002915	272	−0.141	[−0.274–0.008]	**0.039**	0.453	0.542	0.08	S Shelf	chr4:149369175
				cg05483455	272	2.072	[0.401–3.742]	**0.016**	0.370	0.929	0.01	Open Sea	chr5:142762613
				cg05900547	272	0.743	[0.133–1.353]	**0.018**	0.370	0.824	0.02	Open Sea	chr5:142769791
				cg21702128	272	−1.226	[−2.284–0.167]	**0.024**	0.370	0.082	0.01	CpG Island	chr5:142784721
				cg13764763	272	−0.244	[−0.442–0.046]	**0.017**	0.370	0.854	0.06	S Shore	chr5:142785501
				cg23776787	272	−1.234	[−2.401–0.066]	**0.039**	0.453	0.052	0.02	Open Sea	chr5:142814315
		**Recognition**	**CS**	cg13157799	303	11.200	[2.307–20.094]	**0.014**	0.646	0.059	0.01	Open Sea	chr4:149191991
				cg24801588	303	−8.602	[−16.923–0.281]	**0.044**	0.646	0.876	0.01	Open Sea	chr5:142689858
				cg12888360	303	3.330	[0.432–6.228]	**0.025**	0.646	0.191	0.03	Open Sea	chr5:142732502
				cg18484679	303	−6.393	[−12.734–0.052]	**0.049**	0.646	0.854	0.02	Open Sea	chr5:142740314
				cg16586394	303	9.182	[0.127–18.236]	**0.048**	0.646	0.909	0.01	Open Sea	chr5:142757011
				cg07742588	303	−3.640	[−6.079–1.201]	**0.004**	0.343	0.789	0.06	N Shore	chr5:142780439
				cg13764763	303	1.747	[0.076–3.419]	**0.041**	0.646	0.854	0.06	S Shore	chr5:142785501
			**Long**	cg07275757	272	0.287	[0.023–0.55]	**0.034**	0.870	0.896	0.01	Open Sea	chr4:149189974
				cg07742588	272	0.086	[0.015–0.157]	**0.019**	0.867	0.789	0.06	N Shore	chr5:142780439
				cg00294552	272	−0.126	[−0.244–0.008]	**0.038**	0.870	0.842	0.02	N Shore	chr5:142780486
				cg23776787	272	−0.402	[−0.692–0.112]	**0.007**	0.654	0.052	0.02	Open Sea	chr5:142814315
	**Brain Imaging**	**Aβ Burden**	**CS**	cg19496136	305	−134.768	[−234.57–34.966]	**0.009**	0.473	0.746	0.06	Open Sea	chr4:149336765
				cg06968181	305	930.028	[224.302–1635.754]	**0.010**	0.473	0.053	0.02	CpG Island	chr5:142784323
				cg13764763	305	71.510	[3.197–139.824]	**0.041**	0.814	0.854	0.06	S Shore	chr5:142785501
				cg23430507	305	203.561	[30.294–376.828]	**0.022**	0.675	0.679	0.04	Open Sea	chr5:142798375
			**Long**	cg07275757	277	55.967	[21.307–90.627]	**0.002**	0.160	0.896	0.01	Open Sea	chr4:149189974
				cg14438279	277	14.214	[1.578–26.85]	**0.028**	0.860	0.807	0.06	Open Sea	chr5:142806343
		**Grey Matter Volume**	**CS**	cg23329208	292	−211.427	[−401.464–21.389]	**0.030**	0.642	0.892	0.01	N Shelf	chr4:149360981
				cg12741214	292	−287.022	[−546.46–27.584]	**0.031**	0.642	0.922	0.01	Open Sea	chr5:142695619
				cg04457787	292	−287.289	[−530.226–44.352]	**0.021**	0.642	0.919	0.01	Open Sea	chr5:142695636
				cg19645279	292	68.958	[5.299–132.617]	**0.035**	0.642	0.506	0.03	Open Sea	chr5:142702733
				cg16535116	292	−107.983	[−211.523–4.443]	**0.042**	0.642	0.824	0.02	Open Sea	chr5:142769612
				cg14438279	292	−72.913	[−141.02–4.805]	**0.037**	0.642	0.807	0.06	Open Sea	chr5:142806343
			**Long**	cg16219186	126	−15.086	[−25.662–4.509]	**0.006**	0.281	0.885	0.02	N Shore	chr5:142780531
				cg01967637	126	−15.296	[−29.515–1.078]	**0.037**	0.656	0.058	0.01	CpG Island	chr5:142784019
				cg06968181	126	−36.481	[−71.867–1.095]	**0.046**	0.656	0.053	0.02	CpG Island	chr5:142784323
				cg21702128	126	20.044	[2.742–37.346]	**0.025**	0.656	0.082	0.01	CpG Island	chr5:142784721
				cg23776787	126	−31.819	[−50.702–12.936]	**0.001**	0.119	0.052	0.02	Open Sea	chr5:142814315
		**Hippocampal Volume**	**CS**	cg17253842	292	2.314	[0.161–4.466]	**0.036**	0.876	0.871	0.02	Open Sea	chr4:149347991
				cg25708981	292	−1.371	[−2.258–0.485]	**0.003**	0.244	0.811	0.05	Open Sea	chr5:142697868
			**Long**	cg00328411	126	−0.376	[−0.726–0.026]	**0.038**	0.531	0.907	0.01	Open Sea	chr4:149205617
				cg27225476	126	−0.235	[−0.399–0.071]	**0.006**	0.483	0.859	0.02	Open Sea	chr4:149275542
				cg16535116	126	0.138	[0.006–0.269]	**0.043**	0.531	0.824	0.02	Open Sea	chr5:142769612
				cg16219186	126	−0.195	[−0.351–0.038]	**0.016**	0.483	0.885	0.02	N Shore	chr5:142780531
				cg08845721	126	−0.180	[−0.331–0.029]	**0.021**	0.483	0.903	0.02	N Shore	chr5:142780693
				cg21702128	126	0.316	[0.065–0.566]	**0.015**	0.483	0.082	0.01	CpG Island	chr5:142784721
		**Ventricular Volume**	**CS**	cg18484679	292	127.466	[15.907–239.025]	**0.026**	0.939	0.854	0.02	Open Sea	chr5:142740314
				cg16535116	292	92.112	[7.127–177.098]	**0.035**	0.939	0.824	0.02	Open Sea	chr5:142769612
			**Long**	cg27234800	126	−3.390	[−5.982–0.798]	**0.012**	0.468	0.891	0.05	Open Sea	chr4:149116611
				cg18718518	126	−4.607	[−7.848–1.367]	**0.006**	0.468	0.533	0.05	S Shore	chr5:142785455
				cg13764763	126	4.258	[0.872–7.644]	**0.015**	0.468	0.854	0.06	S Shore	chr5:142785501
		**White Matter Volume**	**CS**	cg23650353	292	−231.535	[−461.172–1.898]	**0.049**	0.533	0.921	0.03	Open Sea	chr4:148993643
				cg25672354	292	−165.087	[−325.749–4.424]	**0.045**	0.533	0.867	0.02	Open Sea	chr4:149122383
				cg09238384	292	253.524	[14.776–492.272]	**0.038**	0.533	0.880	0.01	Open Sea	chr4:149358199
				cg15374100	292	280.045	[51.49–508.6]	**0.017**	0.391	0.870	0.01	Open Sea	chr5:142651239
				cg15740681	292	410.969	[103.37–718.568]	**0.009**	0.286	0.891	0.01	Open Sea	chr5:142653066
				cg19820298	292	57.708	[8.645–106.77]	**0.022**	0.403	0.783	0.06	Open Sea	chr5:142770782
				cg07742588	292	112.161	[40.406–183.916]	**0.002**	0.184	0.789	0.06	N Shore	chr5:142780439
				cg23776787	292	417.311	[135.506–699.115]	**0.004**	0.184	0.052	0.02	Open Sea	chr5:142814315
			**Long**	cg13157799	126	13.774	[1.172–26.375]	**0.034**	0.838	0.059	0.01	Open Sea	chr4:149191991
				cg05483455	126	−16.886	[−32.871–0.901]	**0.041**	0.838	0.929	0.01	Open Sea	chr5:142762613
				cg01967637	126	−11.344	[−20.154–2.534]	**0.013**	0.600	0.058	0.01	CpG Island	chr5:142784019
				cg06968181	126	−30.780	[−52.696–8.864]	**0.007**	0.600	0.053	0.02	CpG Island	chr5:142784323
**ADNI**	**Cognition**	**PACC**	**CS**	cg19496136	101	2.324	[0.741–3.906]	**0.005**	0.520	0.660	0.07	Open Sea	chr4:149336765
				cg00407401	101	3.431	[0.457–6.405]	**0.026**	0.677	0.827	0.04	Open Sea	chr5:142690959
				cg19457823	101	3.380	[0.509–6.25]	**0.023**	0.677	0.797	0.05	Open Sea	chr5:142692961
				cg27122725	101	−2.445	[−4.365–0.524]	**0.015**	0.677	0.188	0.06	N Shore	chr5:142781723
			**Long**	cg16594263	93	0.982	[0.113–1.851]	**0.030**	0.902	0.912	0.01	Open Sea	chr5:142768048
				cg19432243	93	−0.090	[−0.163–0.017]	**0.018**	0.902	0.542	0.11	Open Sea	chr5:142770757
				cg00294552	93	−0.178	[−0.319–0.037]	**0.016**	0.902	0.766	0.05	N Shore	chr5:142780486
				cg18849621	93	0.554	[0.01–1.098]	**0.049**	0.902	0.068	0.01	CpG Island	chr5:142784382
	**Brain Imaging**	**Aβ Burden**	**CS**	cg08264907	102	338.502	[17.132–659.871]	**0.042**	0.910	0.861	0.02	Open Sea	chr4:149144045
				cg27107893	102	−67.104	[−121.854–12.353]	**0.018**	0.910	0.593	0.13	Open Sea	chr5:142776274
				cg12969488	102	150.568	[2.892–298.243]	**0.049**	0.910	0.767	0.05	N Shore	chr5:142780984
				cg23430507	102	229.456	[19.698–439.214]	**0.035**	0.910	0.725	0.05	Open Sea	chr5:142798375
			**Long**	cg21701890	93	19.658	[0.888–38.428]	**0.043**	0.564	0.624	0.03	Open Sea	chr4:149094632
				cg27225476	93	−12.817	[−24.37–1.264]	**0.033**	0.564	0.774	0.05	Open Sea	chr4:149275542
				cg19496136	93	−10.671	[−20.315–1.027]	**0.033**	0.564	0.660	0.07	Open Sea	chr4:149336765
				cg15374100	93	−18.402	[−33.893–2.912]	**0.022**	0.564	0.809	0.03	Open Sea	chr5:142651239
				cg19457823	93	−18.606	[−35.806–1.406]	**0.037**	0.564	0.797	0.05	Open Sea	chr5:142692961
				cg24052866	93	12.517	[0.702–24.331]	**0.041**	0.564	0.759	0.05	Open Sea	chr5:142727470
				cg22233604	93	27.999	[3.435–52.562]	**0.028**	0.564	0.844	0.03	Open Sea	chr5:142729377
				cg01967637	93	−30.397	[−59.479–1.315]	**0.044**	0.564	0.065	0.02	CpG Island	chr5:142784019
		**Grey Matter Volume**	**CS**	cg13000004	101	162.787	[29.75–295.825]	**0.019**	0.322	0.820	0.03	Open Sea	chr4:149074093
				cg00328411	101	141.984	[12.584–271.384]	**0.034**	0.392	0.854	0.03	Open Sea	chr4:149205617
				cg13996731	101	−372.339	[−680.468–64.21]	**0.020**	0.322	0.052	0.01	CpG Island	chr4:149363522
				cg11239219	101	−319.257	[−560.259–78.255]	**0.011**	0.322	0.062	0.03	CpG Island	chr4:149365887
				cg13514002	101	579.720	[108.339–1051.101]	**0.018**	0.322	0.916	0.01	Open Sea	chr5:142677292
				cg19645279	101	−78.384	[−146.067–10.7]	**0.026**	0.330	0.525	0.05	Open Sea	chr5:142702733
				cg19432243	101	52.759	[18.152–87.365]	**0.004**	0.322	0.542	0.11	Open Sea	chr5:142770757
				cg08818984	101	−369.293	[−634.387–104.199]	**0.008**	0.322	0.067	0.02	Open Sea	chr5:142814827
				cg11022710	101	−368.042	[−677.198–58.887]	**0.022**	0.322	0.911	0.01	Open Sea	chr5:142820479
			**Long**	cg27107893	91	2.670	[0.129–5.211]	**0.043**	0.866	0.593	0.13	Open Sea	chr5:142776274
				cg12969488	91	−6.927	[−13.593–0.261]	**0.045**	0.866	0.767	0.05	N Shore	chr5:142780984
				cg07528216	91	−32.336	[−58.359–6.312]	**0.017**	0.866	0.902	0.01	S Shelf	chr5:142788776
				cg21979215	91	−3.395	[−6.5–0.29]	**0.035**	0.866	0.624	0.10	Open Sea	chr5:142815807
				cg11022710	91	33.659	[3.272–64.047]	**0.033**	0.866	0.911	0.01	Open Sea	chr5:142820479
		**Hippocampal Volume**	**CS**	cg08264907	101	4.012	[1.264–6.761]	**0.005**	0.272	0.861	0.02	Open Sea	chr4:149144045
				cg27225476	101	1.958	[0.682–3.233]	**0.003**	0.272	0.774	0.05	Open Sea	chr4:149275542
				cg06240648	101	3.751	[0.903–6.599]	**0.012**	0.296	0.861	0.03	Open Sea	chr4:149289762
				cg09238384	101	2.828	[0.348–5.308]	**0.028**	0.360	0.832	0.03	Open Sea	chr4:149358199
				cg23273257	101	8.120	[1.226–15.014]	**0.023**	0.360	0.922	0.01	Open Sea	chr5:142658828
				cg26081259	101	5.790	[0.863–10.718]	**0.024**	0.360	0.909	0.01	Open Sea	chr5:142659682
				cg07715663	101	3.661	[0.184–7.138]	**0.042**	0.481	0.863	0.02	Open Sea	chr5:142721796
				cg16535116	101	1.866	[0.483–3.249]	**0.010**	0.296	0.741	0.05	Open Sea	chr5:142769612
				cg08845721	101	4.192	[0.543–7.841]	**0.027**	0.360	0.887	0.02	N Shore	chr5:142780693
			**Long**	cg00328411	91	−0.200	[−0.366–0.033]	**0.021**	0.641	0.854	0.03	Open Sea	chr4:149205617
				cg12841684	91	−0.404	[−0.734–0.074]	**0.019**	0.641	0.908	0.01	Open Sea	chr4:149251768
				cg27225476	91	−0.102	[−0.195–0.009]	**0.036**	0.641	0.774	0.05	Open Sea	chr4:149275542
				cg19496136	91	−0.099	[−0.184–0.014]	**0.026**	0.641	0.660	0.07	Open Sea	chr4:149336765
				cg07715663	91	−0.256	[−0.502–0.01]	**0.045**	0.641	0.863	0.02	Open Sea	chr5:142721796
				cg16535116	91	−0.106	[−0.209–0.003]	**0.046**	0.641	0.741	0.05	Open Sea	chr5:142769612
				cg13648501	91	0.306	[0.023–0.59]	**0.037**	0.641	0.073	0.02	S Shore	chr5:142785258
		**Ventricular Volume**	**CS**	cg07335874	101	441.994	[46.292–837.695]	**0.031**	0.429	0.908	0.01	Open Sea	chr4:149136507
				cg19650300	101	−145.714	[−278.281–13.147]	**0.034**	0.429	0.824	0.03	Open Sea	chr4:149357960
				cg25708981	101	−63.053	[−112.795–13.31]	**0.015**	0.429	0.704	0.07	Open Sea	chr5:142697868
				cg19645279	101	123.211	[54.555–191.867]	**0.001**	0.071	0.525	0.05	Open Sea	chr5:142702733
				cg05900547	101	−76.113	[−140.852–11.375]	**0.024**	0.429	0.730	0.06	Open Sea	chr5:142769791
				cg19820298	101	−33.578	[−65.412–1.744]	**0.042**	0.429	0.604	0.13	Open Sea	chr5:142770782
				cg12969488	101	78.617	[4.793–152.441]	**0.040**	0.429	0.767	0.05	N Shore	chr5:142780984
				cg26464411	101	−155.611	[−290.419–20.802]	**0.026**	0.429	0.096	0.03	CpG Island	chr5:142784222
				cg21702128	101	277.206	[7.84–546.572]	**0.047**	0.438	0.073	0.01	CpG Island	chr5:142784721
				cg13648501	101	246.583	[36.187–456.979]	**0.024**	0.429	0.073	0.02	S Shore	chr5:142785258
				cg01751279	101	−271.567	[−487.635–55.499]	**0.016**	0.429	0.836	0.03	Open Sea	chr5:142793924
			**Long**	cg07528216	91	35.917	[16.606–55.228]	**0.000**	**0.050**	0.902	0.01	S Shelf	chr5:142788776
				cg16224829	91	10.208	[0.343–20.074]	**0.046**	0.995	0.826	0.03	Open Sea	chr5:142792698
				cg11022710	91	−28.068	[−51.102–5.034]	**0.019**	0.995	0.911	0.01	Open Sea	chr5:142820479
		**White Matter Volume**	**CS**	cg23650353	101	−749.850	[−1303.509–196.19]	**0.009**	0.668	0.930	0.02	Open Sea	chr4:148993643
				cg21701890	101	211.276	[35.537–387.016]	**0.021**	0.668	0.624	0.03	Open Sea	chr4:149094632
				cg27107893	101	−52.397	[−93.036–11.758]	**0.013**	0.668	0.593	0.13	Open Sea	chr5:142776274
			**Long**	cg19432243	91	−2.670	[−4.875–0.464]	**0.020**	0.929	0.542	0.11	Open Sea	chr5:142770757

Linear regression results of nominally significant associations between CpG sites and brain-health phenotypes analysed in both the cross-sectional and long setting in AIBL and ADNI cohorts. The coefficient is represented as β, the p value represents the significance of the CpG methylation status with the outcome interest and the q value is the FDR corrected p value. Mean methylation refers to the average methylation levels at that site for the cohort, along with the sta deviation (S.D.). Covariates: Baseline Age, Intercept Trait Value (when assessed longitudinally), Sex, *APOE-ε4*, Education, Smoking Status, Cellular Composite CD4T, CD8T, eosinophil, monocyte, neutrophil, natural killer cells). Abbreviations: AIBL, Australian Imaging Biomarker and Lifestyle Study; ADNI, Alzheimer’s D Neuroimaging Initiative; CS, cross-sectional; Long, longitudinal; FDR, false discovery rate; PACC, Pre-Clinical Alzheimer’s disease Cognitive Composite; S.D. s deviation.

**Table 3:** FDR Significant associations between stress-related trait x CpG interaction term and Brain-health phenotypes.

Study	AD Trait			Stress Measure	CpG Site	n	p	q	Mean Methylation	SD	Methylation Range	Region	Location Build37
**AIBL**	**Cognition**	**PACC**	**Long**	GDS	cg10590842	268	**0.0003**	**0.0142**	0.903	0.01	0.10	Open Sea	chr4:149083955
				GDS	cg24052866	268	**< 0.0001**	**0.0003**	0.693	0.04	0.44	Open Sea	chr5:142727470
		**Episodic Recall**	**Long**	GDS	cg24052866	268	**0.0002**	**0.0144**	0.693	0.04	0.44	Open Sea	chr5:142727470
		**Language**	**Long**	GDS	cg09143276	268	**0.0009**	**0.0402**	0.808	0.02	0.13	Open Sea	chr4:149066518
				GDS	cg24052866	268	**0.0001**	**0.0055**	0.693	0.04	0.44	Open Sea	chr5:142727470
	**Brain Imaging**	**Grey Matter Volume**	**CS**	HADS-D	cg11239219	288	**0.0003**	**0.0317**	0.055	0.02	0.37	CpG Island	chr4:149365887
			**Long**	HADS-D	cg10590842	124	**0.0010**	**0.0227**	0.903	0.01	0.10	Open Sea	chr4:149083955
				HADS-D	cg21701890	124	**0.0070**	**0.0492**	0.616	0.03	0.23	Open Sea	chr4:149094632
				HADS-D	cg04867484	124	**0.0086**	**0.0492**	0.062	0.03	0.60	Open Sea	chr4:149297469
				HADS-D	cg09238384	124	**0.0065**	**0.0492**	0.880	0.01	0.10	Open Sea	chr4:149358199
				HADS-D	cg05437692	124	**0.0066**	**0.0492**	0.288	0.04	0.32	N Shore	chr4:149362435
				HADS-D	cg12741214	124	**0.0076**	**0.0492**	0.922	0.01	0.05	Open Sea	chr5:142695619
				HADS-D	cg20728768	124	**0.0019**	**0.0339**	0.285	0.06	0.42	Open Sea	chr5:142696594
				HADS-D	cg25708981	124	**0.0003**	**0.0174**	0.811	0.05	0.94	Open Sea	chr5:142697868
				HADS-D	cg03857453	124	**0.0048**	**0.0492**	0.718	0.05	0.42	Open Sea	chr5:142729913
				HADS-D	cg15115787	124	**0.0057**	**0.0492**	0.233	0.05	0.34	Open Sea	chr5:142730701
				HADS-D	cg19820298	124	**0.0022**	**0.0339**	0.783	0.06	0.31	Open Sea	chr5:142770782
				HADS-D	cg12969488	124	**0.0085**	**0.0492**	0.696	0.04	0.36	N Shore	chr5:142780984
				HADS-D	cg01751279	124	**0.0055**	**0.0492**	0.839	0.04	0.36	Open Sea	chr5:142793924
				HADS-D	cg14438279	124	**0.0077**	**0.0492**	0.807	0.06	0.47	Open Sea	chr5:142806343
				HADS-D	cg08423118	124	**0.0010**	**0.0227**	0.775	0.07	0.32	Open Sea	chr5:142808610
				HADS-D	cg03906910	124	**0.0004**	**0.0174**	0.221	0.05	0.38	Open Sea	chr5:142814388
			**CS**	GDS	cg25672354	288	**< 0.0001**	**0.0008**	0.867	0.02	0.24	Open Sea	chr4:149122383
		**Hippocampal Volume**	**Long**	HADS-A	cg27460943	124	**0.0005**	**0.0436**	0.083	0.03	0.31	N Shore	chr4:149362809
				HADS-A	cg01751279	124	**0.0011**	**0.0494**	0.839	0.04	0.36	Open Sea	chr5:142793924
			**CS**	HADS-D	cg03746860	288	**0.0003**	**0.0276**	0.895	0.01	0.14	Open Sea	chr5:142759375
			**Long**	HADS-D	cg19491599	124	**0.0023**	**0.0159**	0.845	0.02	0.21	Open Sea	chr4:149000607
				HADS-D	cg10993059	124	**0.0028**	**0.0175**	0.892	0.01	0.25	Open Sea	chr4:149123062
				HADS-D	cg07335874	124	**0.0002**	**0.0039**	0.897	0.02	0.25	Open Sea	chr4:149136507
				HADS-D	cg00328411	124	**< 0.0001**	**0.0016**	0.907	0.01	0.11	Open Sea	chr4:149205617
				HADS-D	cg04867484	124	**0.0021**	**0.0158**	0.062	0.03	0.60	Open Sea	chr4:149297469
				HADS-D	cg19650300	124	**0.0080**	**0.0368**	0.872	0.01	0.13	Open Sea	chr4:149357960
				HADS-D	cg05437692	124	**< 0.0001**	**0.0009**	0.288	0.04	0.32	N Shore	chr4:149362435
				HADS-D	cg27460943	124	**0.0006**	**0.0076**	0.083	0.03	0.31	N Shore	chr4:149362809
				HADS-D	cg19457823	124	**0.0017**	**0.0145**	0.853	0.05	0.45	Open Sea	chr5:142692961
				HADS-D	cg12741214	124	**0.0008**	**0.0086**	0.922	0.01	0.05	Open Sea	chr5:142695619
				HADS-D	cg25708981	124	**0.0003**	**0.0048**	0.811	0.05	0.94	Open Sea	chr5:142697868
				HADS-D	cg03857453	124	**0.0002**	**0.0039**	0.718	0.05	0.42	Open Sea	chr5:142729913
				HADS-D	cg15115787	124	**0.0065**	**0.0314**	0.233	0.05	0.34	Open Sea	chr5:142730701
				HADS-D	cg16586394	124	**0.0043**	**0.0249**	0.909	0.01	0.20	Open Sea	chr5:142757011
				HADS-D	cg25535999	124	**0.0057**	**0.0290**	0.894	0.01	0.09	Open Sea	chr5:142757312
				HADS-D	cg27107893	124	**0.0055**	**0.0290**	0.793	0.10	0.94	Open Sea	chr5:142776274
				HADS-D	cg01751279	124	**0.0107**	**0.0446**	0.839	0.04	0.36	Open Sea	chr5:142793924
				HADS-D	cg23430507	124	**0.0104**	**0.0446**	0.679	0.04	0.32	Open Sea	chr5:142798375
				HADS-D	cg14438279	124	**0.0001**	**0.0029**	0.807	0.06	0.47	Open Sea	chr5:142806343
				HADS-D	cg08423118	124	**0.0008**	**0.0086**	0.775	0.07	0.32	Open Sea	chr5:142808610
				HADS-D	cg23776787	124	**0.0026**	**0.0171**	0.052	0.02	0.42	Open Sea	chr5:142814315
				HADS-D	cg03906910	124	**0.0010**	**0.0087**	0.221	0.05	0.38	Open Sea	chr5:142814388
			**Long**	GDS	cg13996731	124	**< 0.0001**	**0.0004**	0.059	0.01	0.13	CpG Island	chr4:149363522
		**Ventricular Volume**	**Long**	HADS-A	cg25672354	124	**0.0012**	**0.0425**	0.867	0.02	0.24	Open Sea	chr4:149122383
				HADS-A	cg10993059	124	**0.0014**	**0.0425**	0.892	0.01	0.25	Open Sea	chr4:149123062
				HADS-A	cg12888360	124	**0.0010**	**0.0425**	0.191	0.03	0.22	Open Sea	chr5:142732502
			**Long**	HADS-D	cg23650353	124	**0.0090**	**0.0375**	0.921	0.03	0.43	Open Sea	chr4:148993643
				HADS-D	cg09143276	124	**0.0078**	**0.0375**	0.808	0.02	0.13	Open Sea	chr4:149066518
				HADS-D	cg10590842	124	**0.0088**	**0.0375**	0.903	0.01	0.10	Open Sea	chr4:149083955
				HADS-D	cg21701890	124	**0.0004**	**0.0051**	0.616	0.03	0.23	Open Sea	chr4:149094632
				HADS-D	cg27234800	124	**0.0034**	**0.0208**	0.891	0.05	0.39	Open Sea	chr4:149116611
				HADS-D	cg10993059	124	**< 0.0001**	**0.0006**	0.892	0.01	0.25	Open Sea	chr4:149123062
				HADS-D	cg07335874	124	**< 0.0001**	**0.0006**	0.897	0.02	0.25	Open Sea	chr4:149136507
				HADS-D	cg04867484	124	**0.0005**	**0.0053**	0.062	0.03	0.60	Open Sea	chr4:149297469
				HADS-D	cg27460943	124	**0.0051**	**0.0275**	0.083	0.03	0.31	N Shore	chr4:149362809
				HADS-D	cg13996731	124	**0.0021**	**0.0146**	0.059	0.01	0.13	CpG Island	chr4:149363522
				HADS-D	cg15374100	124	**0.0021**	**0.0146**	0.870	0.01	0.21	Open Sea	chr5:142651239
				HADS-D	cg15740681	124	**0.0022**	**0.0146**	0.891	0.01	0.10	Open Sea	chr5:142653066
				HADS-D	cg19457823	124	**0.0087**	**0.0375**	0.853	0.05	0.45	Open Sea	chr5:142692961
				HADS-D	cg12741214	124	**0.0014**	**0.0117**	0.922	0.01	0.05	Open Sea	chr5:142695619
				HADS-D	cg20728768	124	**0.0050**	**0.0275**	0.285	0.06	0.42	Open Sea	chr5:142696594
				HADS-D	cg25708981	124	**0.0083**	**0.0375**	0.811	0.05	0.94	Open Sea	chr5:142697868
				HADS-D	cg19645279	124	**0.0001**	**0.0016**	0.506	0.03	0.23	Open Sea	chr5:142702733
				HADS-D	cg12888360	124	**0.0014**	**0.0117**	0.191	0.03	0.22	Open Sea	chr5:142732502
				HADS-D	cg01967637	124	**0.0001**	**0.0015**	0.058	0.01	0.09	CpG Island	chr5:142784019
				HADS-D	cg14438279	124	**0.0008**	**0.0085**	0.807	0.06	0.47	Open Sea	chr5:142806343
				HADS-D	cg23776787	124	**< 0.0001**	**0.0002**	0.052	0.02	0.42	Open Sea	chr5:142814315
				HADS-D	cg03906910	124	**0.0002**	**0.0032**	0.221	0.05	0.38	Open Sea	chr5:142814388
			**Long**	GDS	cg13000004	124	**0.0017**	**0.0382**	0.870	0.01	0.10	Open Sea	chr4:149074093
				GDS	cg27234800	124	**0.0013**	**0.0382**	0.891	0.05	0.39	Open Sea	chr4:149116611
				GDS	cg10993059	124	**< 0.0001**	**0.0008**	0.892	0.01	0.25	Open Sea	chr4:149123062
				GDS	cg13996731	124	**0.0003**	**0.0118**	0.059	0.01	0.13	CpG Island	chr4:149363522
		**White Matter Volume**	**Long**	GDS	cg20598211	124	**0.0002**	**0.0140**	0.916	0.01	0.21	Open Sea	chr5:142762454
**ADNI**	**Brain Imaging**	**Aβ Burden**	**CS**	GDS	cg12888360	102	**0.0002**	**0.0171**	0.260	0.05	0.36	Open Sea	chr5:142732502
		**Grey Matter Volume**	**CS**	GDS	cg07589972	101	**0.0003**	**0.0336**	0.867	0.02	0.15	Open Sea	chr5:142815463
			**Long**	GDS	cg04867484	91	**0.0007**	**0.0227**	0.070	0.04	0.59	Open Sea	chr4:149297469
				GDS	cg19135245	91	**0.0001**	**0.0032**	0.068	0.02	0.15	CpG Island	chr5:142784187
				GDS	cg21979215	91	**< 0.0001**	**0.0019**	0.624	0.10	0.65	Open Sea	chr5:142815807
		**Hippocampal Volume**	**CS**	GDS	cg05437692	101	**0.0004**	**0.0450**	0.296	0.05	0.45	N Shore	chr4:149362435
			**Long**	GDS	cg08845721	91	**0.0010**	**0.0498**	0.887	0.02	0.15	N Shore	chr5:142780693
				GDS	cg21979215	91	**0.0007**	**0.0498**	0.624	0.10	0.65	Open Sea	chr5:142815807
		**Ventricular Volume**	**Long**	GDS	cg13996731	91	**0.0002**	**0.0049**	0.052	0.01	0.12	CpG Island	chr4:149363522
				GDS	cg24801588	91	**< 0.0001**	**0.0002**	0.835	0.02	0.27	Open Sea	chr5:142689858
				GDS	cg25708981	91	**0.0021**	**0.0367**	0.704	0.07	0.59	Open Sea	chr5:142697868
				GDS	cg07528216	91	**0.0001**	**0.0021**	0.902	0.01	0.12	S Shelf	chr5:142788776
				GDS	cg07589972	91	**0.0015**	**0.0314**	0.867	0.02	0.15	Open Sea	chr5:142815463
				GDS	cg21979215	91	**< 0.0001**	**0.0002**	0.624	0.10	0.65	Open Sea	chr5:142815807

Linear regression results of stress-trait x CpG site interaction terms with Brain-health phenotypes that reached FDR significance. The p value represents the of the interaction term with the outcome trait of interest, whilst the q value represents the FDR corrected p value. Those p and q values that reached < 0.05 a Covariates: Baseline Age, Intercept Trait Value (when assessed longitudinally), Sex, *APOE-ε4*, Education, Smoking Status, Cellular Composition (B cell, CD4T, eosinophil, monocyte, neutrophil, natural killer cells). Abbreviations: AIBL, Australian Imaging Biomarker and Lifestyle Study; ADNI, Alzheimer’s Disease Neur Initiative; CS, cross-sectional; Long, longitudinal; FDR, false discovery rate; GDS, Geriatric Depression Scale; HADS, Hospital Anxiety and Depression Scale; PA clinical Alzheimer’s disease Cognitive Composite.
